# Axillary fibroadenoma: A rare case report and review of literature

**DOI:** 10.1016/j.radcr.2026.06.150

**Published:** 2026-07-21

**Authors:** Abdulwahid M. Salih, Lana R.A. Pshtiwan, Ari M. Abdullah, Hiwa O. Baba, Rebaz O. Mohammed, Sakar O. Arif, Berun A. Abdalla, Masty K. Ahmed, Sara N. Ahmad, Fahmi H. Kakamad

**Affiliations:** a Department of Breast Surgery, Smart Health Tower, Sulaymaniyah, Iraq; b Department of Radiology, Smart Health Tower, Sulaymaniyah, Iraq; c Department of Pathology, Sulaymaniyah Teaching Hospital, Sulaymaniyah, Iraq; d Department of Pathology, Smart Health Tower, Sulaymaniyah, Iraq; e Department of General Surgery, Smart Health Tower, Sulaymaniyah, Iraq; f Department of Scientific Affairs, Smart Health Tower, Sulaymaniyah, Iraq; g Kscien Organization for Scientific Research (Middle East Office), Sulaymaniyah, Iraq; h Department of Clinical Sciences, College of Medicine, University of Sulaimani, Sulaymaniyah, Iraq

**Keywords:** Axillary fibroadenoma, Ectopic breast tissue, Accessory breast, Benign breast tumor, Surgical excision

## Abstract

Fibroadenoma (FA) is a common benign tumor of the breast, but its occurrence in the axillary region is exceptionally rare. Such lesions may arise from accessory breast tissue or develop de novo within axillary stromal tissue, often mimicking lymphadenopathy or other soft-tissue masses. This report presents a rare axillary FA in a young female, highlighting its unusual site and diagnostic importance. A 23-year-old woman presented with a painless swelling in the left axilla that had gradually increased in size over 5 months. Ultrasound showed an oval, heterogeneously hypoechoic axillary mass with macrolobulated and angular margins, categorized as BI-RADS 3. Ultrasound-guided core needle biopsy and subsequent excision confirmed a fibroadenoma arising from accessory breast tissue, with an uneventful postoperative course and no recurrence at 17 months. A review of the published literature identified 13 cases of axillary fibroadenoma across 12 reports, in women aged 16-41 years. Most were premenopausal (76.9%), right-sided lesions predominated (46.2%), and ultrasound was the most common imaging modality (69.2%). Histopathology confirmed fibroadenoma in all cases, the majority arising from accessory breast tissue, and surgical excision was the usual treatment, with no reported recurrences. Axillary FA is a rare benign lesion of young women. Despite its resemblance to other axillary masses, histopathological confirmation is crucial, and complete excision ensures an excellent prognosis with minimal recurrence risk. On ultrasound, an axillary fibroadenoma can closely mimic a lymph node, so recognition of this appearance is essential to reach an accurate diagnosis and avoid unnecessary intervention.

## Background

Breast development begins early in embryogenesis with ectodermal thickening. By the sixth week of gestation, paired mammary ridges, or milk lines, appear as elongated ectodermal bands extending bilaterally from the axilla to the groin [1]. Normally, these ridges regress most of their length, except for the paired pectoral segments that form the breasts. Incomplete regression leads to the persistence of additional mammary tissue, termed accessory, supernumerary, or ectopic breast (polymastia), typically along the original milk line [2]. Its incidence ranges from 1% to 6% in women and about 1.68% in men [3,4]. The axilla is the most frequent site, accounting for approximately 60%-70% of reported cases [5]. Although pathological involvement of accessory breast (AB) tissue is uncommon, it may occur because the ectopic tissue contains stromal and epithelial components identical to those of the normal breast. Recognition of such tissue is clinically important, as it is susceptible to the same benign and malignant processes that affect the normal mammary gland [6,7].

Fibroadenoma (FA) is a benign biphasic tumor composed of stromal and epithelial elements, most common in women of reproductive age [6]. While it usually arises within the breast parenchyma, its occurrence in the axillary region is exceedingly rare. When present, axillary FA may originate from fibroglandular extensions of the breast or develop de novo within axillary stromal tissue. Because the axilla is not a typical site of mammary proliferation, such lesions may be misinterpreted as lymphadenopathy or other soft-tissue tumors. Therefore, recognizing FA in the axilla, particularly in young females, is essential to avoid unnecessary procedures and to enhance understanding of this unusual localization [8].

This report presents a rare axillary FA in a young female, highlighting its unusual site and diagnostic importance. The case was prepared in accordance with the CaReL guidelines, and all cited references were critically reviewed for eligibility [9,10].

## Case presentation

### Patient information

A 23-year-old unmarried woman with regular menstrual cycles presented with a painless swelling in the left axilla that had been gradually increasing over the past 5 months. She denied any history of trauma, infection, or previous breast or axillary surgery. Her medical, surgical, and family histories were unremarkable.

### Clinical findings

On physical examination, a well-defined, mobile, nontender mass was palpable in the left axillary region. The overlying skin showed no erythema, dimpling, or tethering, and there was no nipple retraction or discharge. Examination of both breasts was otherwise unremarkable, with no palpable masses or lymphadenopathy on the contralateral side.

### Diagnostic approach

**Fig. 1:**
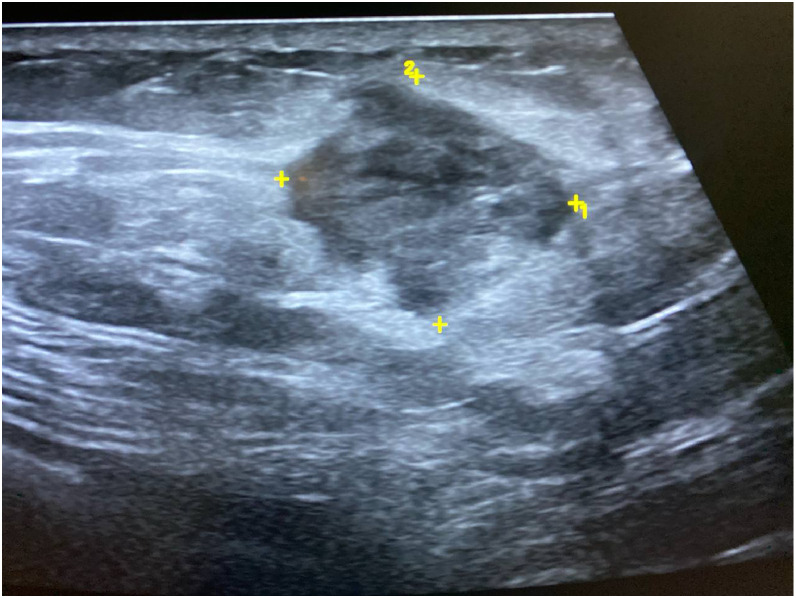
Ultrasound of the left axilla showing an oval, heterogeneously hypoechoic mass oriented parallel to the skin and lying 3-4 mm deep to it. The margins are macrolobulated with an angular upper margin, and no posterior acoustic features are seen. A central echogenic line is present within the lesion. Electronic calipers mark the lesion dimensions.

On ultrasound, the left axillary lesion was an oval, heterogeneously hypoechoic mass located 3-4 mm deep to the skin and oriented parallel to the skin surface. The margins were macrolobulated with an angular upper margin, and no posterior acoustic features were present. A central echogenic line within the lesion initially raised the possibility of an enlarged lymph node. Taking these features together with interval growth over 5 months and the atypical axillary location, the lesion was categorized as BI-RADS 3 (probably benign), and ultrasound-guided core needle biopsy was performed to establish a definitive diagnosis and exclude nodal pathology (Fig. 1). Two additional nodules were noted in the breast, at the 1 and 4 o’clock positions. Both were well-circumscribed, hypoechoic, and avascular, in keeping with incidental probably benign fibroadenomas, and both have remained stable in size on follow-up, consistent with a benign course.

A core needle biopsy of the axillary mass was performed. Histopathological analysis demonstrated compressed ducts lined by benign ductal epithelium within a fibromyxoid stroma, with focal areas of ductal proliferation features consistent with FA.

### Therapeutic intervention

**Fig. 2:**
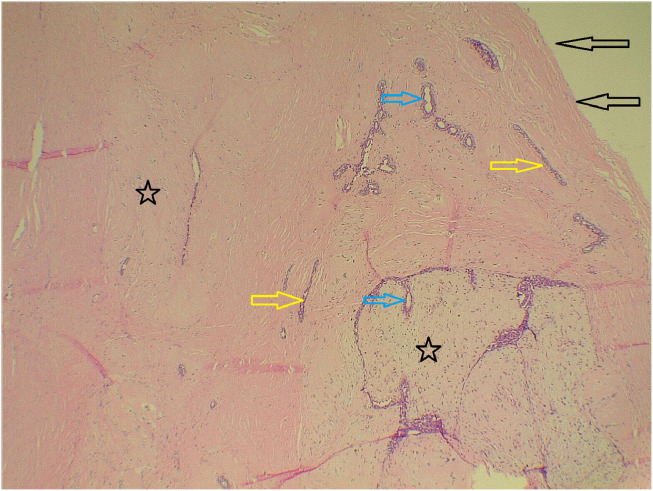
Section reveals a well-defined mass with pushing borders (dark arrows), composed of a mixture of pericanalicular (blue arrows) and intracanalicular (yellow arrows) growth pattern in a fibromyxoid stroma (dark stars). The image was captured using hematoxylin and eosin stain at 4× magnification.

Following multidisciplinary team discussion, surgical excision of the left axillary mass was undertaken under local anesthesia through an elliptical incision. The lesion was completely excised, and the wound was closed in layers. Subsequent histopathological examination of the excised specimen confirmed the diagnosis of FA (Fig. 2). The postoperative course was uneventful, with satisfactory wound healing and no recurrence on follow-up.

### Follow-up and outcome

The postoperative course was smooth, and the surgical wound healed by primary intention without complications. The patient remains asymptomatic and continues regular annual follow-up, with no evidence of recurrence or new lesions at 17 months.

## Discussion

Fibroadenomas are among the most common benign breast tumors, occurring in approximately 25% of women, most frequently between the ages of 15 and 35 years. Although they predominantly arise within the breast parenchyma, fibroadenomas may occasionally develop in extramammary sites such as the axilla or facial region. When located in the axilla, these lesions typically originate from ectopic breast tissue (EBT), which forms due to incomplete regression of the embryonic mammary ridge during development. EBT is present in up to 6% of women, often remaining asymptomatic and easily mistaken for other soft-tissue structures or pathologies, including lipoma or lymphadenopathy, during clinical assessment [11,12].

A non-systematic review of the literature identified 13 cases of axillary fibroadenoma across 12 reports. The patients’ ages ranged from 16-41 years. Ten patients (76.9%) were premenopausal, while 3 (23.1%) had unreported menstrual status. Regarding laterality, 6 (46.2%) lesions occurred on the right side, 3 (23.1%) on the left, 1 (7.7%) was bilateral, and the side was unspecified in 3 (23.1%). Most lesions presented as painless, slowly enlarging masses; 3 patients (23.1%) reported associated pain or discomfort, and 1 (7.7%) was asymptomatic and detected incidentally. Ultrasound was performed in 9 patients (69.2%), typically revealing well-defined, hypoechoic nodules with smooth margins consistent with a benign process, while 1 lesion was characterized by MRI. In all cases, histopathological examination confirmed fibroadenoma, with the majority arising from ectopic or accessory breast tissue. Surgical excision was the treatment of choice in all patients, and, where follow-up was reported, recovery was uneventful with no recurrence [[2], [3], [4], [5], [6], [7], [8], [9], [10], [11], [12], [13], [14], [15], [16], [17]] (Table 1).

**Table 1:** Summary of clinical and demographic characteristics in reported cases of axillary fibroadenoma.

Study	Age (y)	Menstrual status	Presentation	Origin (ectopic vs de novo)	Surgical history	Ultrasound	FNAC	Final diagnosis (histology)	Treatment approach	Outcome
Amaranathan et al. [6]	31	N/A	Right axillary mass, pain and discomfort, 1 y	Ectopic (accessory breast tissue)	N/A	Well-defined hypoechoic lesion and low vascularity	Benign ductal cells consistent with fibroadenoma	Fibroadenoma in ectopic breast tissue	Excision biopsy of subcutaneous lesion	Regular follow-up and no recurrence
Bokhari et al. [15]	16	N/A	Asymptomatic	Ectopic (accessory breast tissue)	N/A	Hypoechoic oval lesion, smooth margin, and posterior enhancement	N/A	Fibroadenoma arising in ectopic breast tissue	Complete surgical excision	Uneventful postoperative recovery 3-mo follow-up; no recurrence
Ciralik et al. [14]	23	N/A	Right axillary mass	Ectopic (accessory breast tissue)	N/A	N/A	Fibroadenoma	Fibroadenoma identical to conventional breast type	Complete surgical excision	N/A
Conde et al. [16]	37	Premenopausal	Right axillary accessory breast nodule	Ectopic (accessory breast tissue)	Tubal ligation	Multiple solid hypoechoic circumscribed masses; right axilla lesion with shadowing + microcalcifications	Fibroadenoma	Fibroadenoma	Surgical excision	No recurrence, 11-mo follow-up
Goyal et al. [4]	23	Premenopausal	Bilateral subcutaneous axillary masses for the last 7 y	Ectopic (accessory breast tissue)	N/A	Soft-tissue masses	N/A	Fibroadenoma in ectopic breast tissue	Surgical excision	Good postoperative recovery and no recurrence
Santosh et al. [13]	28	Premenopausal	Right axillary mass for 2 y	Ectopic (accessory breast tissue)	N/A	Well-defined, smooth, homogeneous hypoechoic lesion; low vascularity	24-G needle nonaspirational method	Fibroadenoma	Surgical excision	Post-operation follow-up of the patients after 6 mo was unremarkable
	20	Premenopausal	Left axillary swelling for 1 y	Ectopic (accessory breast tissue)	N/A	Well-defined, encapsulated, and smooth marginated lesion	24-G needle nonaspirational method	Fibroadenoma	Surgical excision	Postoperation follow-up of the patients after 6 mowas unremarkable
Sawa et al. [5]	41	Premenopausal	Right axillary mass	Ectopic (accessory breast tissue)	N/A	Hypoechoic and distinct margins	N/A	Fibroadenoma arising from axillary accessory breast tissue	Surgical excision	Uneventful recovery
Schonour and Mowry [11]	35	Premenopausal	Palpable right axillary lump for 6-mo	Ectopic (accessory breast tissue)	N/A	Hypoechoic oval mass and smooth margin	N/A	Fibroadenoma arising from axillary accessory breast tissue	Surgical excision	Uneventful postoperative recoveryand no recurrence
Val-Bernal et al. [7]	29	Premenopausal	Mass in the axilla that was associated with discomfort and cosmetic problems	Ectopic (accessory breast tissue)	N/A	N/A	N/A	Fibroadenoma arising from axillary accessory breast tissue	Complete surgical excision	Uneventful recoveryand no recurrence
Virji et al. [8]	37	Premenopausal	Left axillary lump	Ectopic (accessory breast tissue)	incision and drainage for left breast sebaceous cyst	Lobulated hypoechoic solid nodule with vascularity	N/A	Fibroadenoma arising from axillary accessory breast tissue	Surgical excision	Uneventful postoperative recoveryand no recurrence
Yefter and Shibiru [2]	28	Premenopausal	Axillary swelling of 3 y	Ectopic (accessory breast tissue)	N/A	N/A	Fibroadenoma	Fibroadenoma arising from axillary accessory breast tissue	Lumpectomy	Uneventful recoveryand no recurrence after 3 mo follow-up
Ishiguro et al. [17]	36	Premenopausal	Left axillary mass ∼1 y; pain/swelling worse with menses; rapid growth over 40 d	Ectopic (accessory breast tissue)	N/A	MRI: well-defined oval mass ∼10 mm (no US)	N/A	Fibroadenoma from AAB	Complete excision of fibroadenoma + accessory breast	No recurrence at 12 mo

FNAC, fine needle aspiration cytology; N/A, not applicable.

The demographic profile of previously reported cases shows that axillary FA predominantly affects young to middle-aged women, typically between 20 and 40 years [4,14]. Similarly, the present case concerns a 23-year-old woman with a solitary, painless, mobile mass in the left axilla. Unlike most documented cases, which predominantly affect the right axilla [6,15], this lesion developed on the left side. Moreover, while previous reports describe lesions enlarging slowly over 1 to 3 years, this patient experienced a relatively rapid growth over 5 months.

This relatively rapid enlargement may reflect the hormone responsiveness of accessory breast tissue, which, like normally sited breast, contains hormone-sensitive glandular elements that may proliferate in relation to menstrual cyclicity, pregnancy, or lactation. Consistent with this, Ishiguro et al. [17] described an axillary accessory-breast fibroadenoma that progressed from a barely detectable nodule to a clinically obvious mass within approximately 40 days, with symptoms fluctuating across the menstrual cycle [17], illustrating how pronounced this hormonal influence can be. The patient in the present case was a 23-year-old premenopausal woman with regular menstrual cycles, a hormonal context compatible with the relatively rapid 5-month growth observed.

Radiologically, axillary fibroadenomas typically present as well-circumscribed, homogeneous, hypoechoic nodules with smooth margins and minimal vascularity, reflecting their benign nature [4,16]. The differential for an axillary mass is broad, including lymphadenopathy, lipoma, epidermal inclusion cyst, hidradenitis suppurativa, peripheral nerve sheath tumors, phyllodes tumor, and, less commonly, nodal metastasis or lymphoma [2,11]. A useful discriminator is the fatty hilum of a lymph node, which a fibroadenoma lacks [15]; irregular margins, heterogeneous echotexture, or increased Doppler vascularity instead favor malignancy and warrant biopsy [11].

Additional imaging modalities can also play an important role in these cases. Sawa et al. demonstrated that dynamic contrast-enhanced MRI can reveal the accessory breast tissue surrounding an axillary mass and an enhancement pattern typical of fibroadenoma, and that mammography showed only the noncalcified mass with limited additional characterization [5]. Histopathology confirmed the diagnosis by showing compressed ducts lined by benign epithelium within a fibromyxoid stroma, consistent with the microscopic architecture of conventional breast FA [5,14].

While minimally invasive techniques like ultrasound-guided cryoablation offer effective, low-morbidity alternatives for managing benign lesions in the normally situated breast [18], surgical excision remains the treatment of choice for most axillary fibroadenomas, providing both definitive diagnosis and symptom relief. Consistent with previous reports [6,15], the current patient underwent complete local excision with an uneventful postoperative recovery. In contrast, Conde et al. [16] recommended a conservative approach for multiple fibroadenomas involving both breasts and accessory axillary tissue, emphasizing that management should be individualized based on the lesion’s size, number, and clinical impact [16].

Recurrence after surgical excision of axillary fibroadenomas is rare [2,4], and in this case, the patient remains asymptomatic with no evidence of recurrence to date, which aligns with previous findings. The clinical and radiological features of axillary FA can mimic lymphadenopathy or lipoma, potentially delaying diagnosis [8]. Therefore, awareness of this rare entity is crucial to prevent unnecessary interventions and ensure accurate diagnosis through imaging and histopathology.

## Conclusion

Axillary FA is a rare benign lesion of young women. Despite its resemblance to other axillary masses on imaging, histopathological confirmation is crucial, and complete excision ensures an excellent prognosis with minimal recurrence risk.

## Data availability

All data and materials are kept by the first and corresponding authors.

## Patient consent

Written informed consent was obtained from the patient for the participation of the present case report and any accompanying images.
